# Inorganic nitrate, a natural anti-obesity agent: A systematic review and meta-analysis of animal studies

**DOI:** 10.17179/excli2020-2515

**Published:** 2020-07-06

**Authors:** Zahra Bahadoran, Sajad Jeddi, Sevda Gheibi, Parvin Mirmiran, Khosrow Kashfi, Asghar Ghasemi

**Affiliations:** 1 Nutrition and Endocrine Research Center, Research Institute for Endocrine Sciences, Shahid Beheshti University of Medical Sciences, Tehran, Iran; 2 Endocrine Physiology Research Center, Research Institute for Endocrine Sciences, Shahid Beheshti University of Medical Sciences, Tehran, Iran; 3 Department of Clinical Sciences in Malmö, Unit of Molecular Metabolism, Lund University Diabetes Centre, Clinical Research Center, Malmö University Hospital, Lund University, Malmö, Sweden; 4 Department of Clinical Nutrition and Human Dietetics, Faculty of Nutrition Sciences and Food Technology, National Nutrition and Food Technology Research Institute, Shahid Beheshti University of Medical Sciences, Tehran, Iran; 5 Department of Molecular, Cellular and Biomedical Sciences, Sophie Davis School of Biomedical Education, City University of New York School of Medicine, New York, NY 10031, USA

**Keywords:** nitrate, body weight, obesity, rats, systematic review and meta-analysis

## Abstract

Evidence for potential effects of inorganic nitrate (NO_3_) on body weight is limited to inconsistent findings of animal experiments. In this systematic review and meta-analysis, we aimed to quantify the overall effect of inorganic NO_3_, administered via drinking water, on body weight gain in rats. We searched PubMed, Scopus, and Embase databases, and the reference lists of published papers. Experiments on male rats, reported data on body weight in NO_3_-treated animals and controls, were included for quality assessment, meta-analyses, subgroup analyses, and meta-regressions. Of 173 initially obtained studies, 11 were eligible to be included in the analyses, which covered the years 2004 to 2019 and included a total of 43 intervention (n=395) and 43 control (n=395) arms. Overall, the final body weights were significantly lower in the NO_3_-supplemented groups compared to controls (WMD= -16.8 g, 95 % CI= -27.38, -6.24; *P*=0.002). Doses of NO_3_ higher than the median (> 72.94 mg L^-1^ d^-1^) and longer NO_3_ exposure (> 8 weeks) resulted in greater mean differences (WMD= -31.92 g, 95 % CI= -52.90, -10.94 and WMD= -23.16 g, 95 % CI= -35.64, -10.68 g). After exclusion of experiments using high doses of NO_3 _(> 400 mg L^-1^ d^-1^), the overall mean differences in body weights between the groups decreased by approximately 37 % but remained statistically significant (WMD= -10.11 g, 95 % CI= -19.04, -1.19, *P*=0.026). Mean changes in body weight were affected by age, baseline values in body weight, and the duration of the studies. These preliminary experimental findings strongly support the hypothesis that NO_3_ can be considered as a natural anti-obesity agent.

## Introduction

Nitric oxide (NO), a ubiquitous and multifunctional endocrine hormone (Bahadoran and Carlström, 2020[3]), has a critical role in regulation of adipocyte physiology, energy metabolism, physiological control of lipolysis in adipose tissue, and regulation of body composition (Andersson et al., 1999[1]; Sansbury and Hill, 2014[36]). Emerging evidence strongly suggests that decreased NO bioavailability (Gamez-Mendez et al., 2014[8]; Sansbury and Hill, 2014[36]), due to decreased endothelial NO synthase (eNOS) expression/activity, eNOS uncoupling, or increased NO quenching through increased oxidative stress, chronic inflammation or hypoxia, results in adipose tissue dysfunction, which contributes to the development of obesity in both animals and humans (Ghasemi and Jeddi, 2017[9]; Jankovic et al., 2017[19]). 

Since inorganic nitrate (NO_3_) could act as a substrate for endogenous NO generation and exhibits NO-like bioactivity (Lundberg and Govoni, 2004[26]; Lundberg et al., 2008[27]), particularly in the case of diminished eNOS-derived NO, NO_3_ supplementation has been suggested as a promising treatment in management of obesity (Ghasemi and Jeddi, 2017[9]; Lundberg et al., 2018[25]). This hypothesis has not yet been directly addressed in humans due to the conventional perception on the hazardous effects of inorganic NO_3_. Limited animal studies have investigated the anti-obesity effects of inorganic NO_3_ as a primary hypothesis (Roberts et al., 2015[35]), while the majority of the available experiments have only reported data on body weight in NO_3_-treated animals as a secondary crude observation without any significant interpretations. This issue remains even more uncertain since inconsistent results including, higher (Hezel et al., 2016[13]; Oghbaei et al., 2018[32]), lower (Zaki et al., 2004[40]; El-Wakf et al., 2009[6]; Gheibi et al., 2018[10]; Khorasani et al., 2019[22]) or equal (Ashmore et al., 2015[2]; Khalifi et al., 2015[21]; Roberts et al., 2015[35]) weight gain have been reported in control and NO_3_-treated rats. 

Here we, therefore, aimed to quantify the overall effects of inorganic NO_3,_ administered via drinking water, on trends in body weight gain in rats and to identify potential moderators of body weight change in response to orally ingested NO_3_, in the framework of a systematic review and meta-analysis. We also tried to explore the potential source(s) of heterogeneity between the various studies, in order to fulfill an important pillar in meta-analyses involving animal data, which can address defects and limitations of animal experiments and provide new insight into designing new experiments (de Vries et al., 2014[5]; Hooijmans et al., 2014[15]).

## Methods

The Preferred Reporting Items for Systematic Reviews and Meta-Analyses (PRISMA) guidelines were followed during all stages of implementation, analysis, and reporting of this study (Moher et al., 2015[29]).

### Primary exposures and outcomes

The primary exposure of interest was the daily intake of NO_3_ via drinking water, as mg L^-1^ NaNO_3_. The primary outcome of analyses included mean differences in body weights between NO_3_-treated and control groups, the secondary outcome was the mean changes in body weights between NO_3_-treated and control groups, final *vs.* baseline body weight of the animals.

### Search strategy and identification of the studies 

Multiple electronic databases, including PubMed, Scopus, and Embase, were searched for relevant published papers. The primary search was supplemented with hand screening and searching of the citation lists within the papers and then electronic searching using google scholar. Searches were performed without restrictions on years or language through 25 February 2018, and updated through May 2020. A structured search strategy using various combinations of keywords i.e. nitrate, weight, and rat, and Boolean terms were conducted to identify records in each database. 

**Figure 1:**
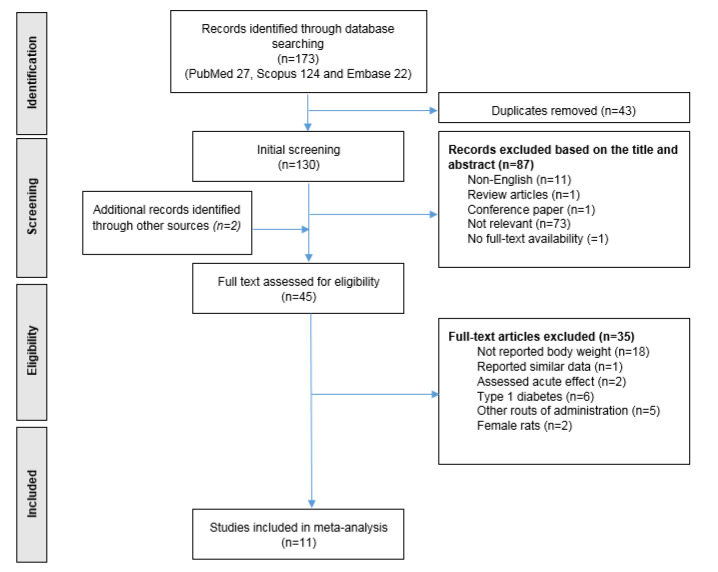
Literature search and screening processes

Since the effect of NO_3_ on body weight was not investigated as a primary outcome, all animal studies that used NO_3_ treatment and reported baseline and final body weights were included in the analyses, regardless of the primary aim of the study. The papers were initially screened using the titles and abstracts; irrelevant records, reviews whether meta-analysis, systematic and narrative, letters to editors and conference papers were excluded. Then, two investigators independently reviewed the full-text records for inclusion and exclusion criteria, and potentially relevant full-text articles were finally retrieved for data extraction. Studies were excluded from the analyses if they did not report baseline and final body weights of the animals, assessed acute effects of NO_3_, single dose of NO_3_ (Kuzenkov and Krushinskii, 2014[24]; Peleli et al., 2015[33]); experiments conducted in female rats were also excluded because of the low number of studies and the sex-difference in body weight gain in rats (Shi and Clegg, 2009[38]). NO_3_-treatment studies that used a different rout of administration other than drinking water (e.g. NO_3_-rich foods) were also excluded. Finally, studies that reported the outcome as mean ± standard deviation (SD) or standard error (SE) per each experimental group, and provided information regarding the number of animals within each group, were included in our meta-analysis. Figure 1(Fig. 1) provides details on the literature search and the screening processes.

### Quality assessment of the studies 

The quality and risk of bias pertaining to the included studies were assessed independently by two investigators (S.J and S.Gh) using the Systematic Review Center for Laboratory Animal Experimentation Risk of Bias Tool (SYRCLE's RoB tool) for animal intervention studies (Hooijmans et al., 2014[16]), with some modifications. In brief, this quality assessment tool is a modified version of the Cochrane RoB tool and includes 10 questions in order to determine potential sources of bias including, selection bias (sequence generation, baseline characteristics, allocation concealment), performance bias (random housing, blinding), detection bias (random outcome assessment and blinding of outcome assessor) attrition bias (incomplete outcome data), and reporting bias (selective outcome reporting) (Hooijmans et al., 2014[16]). In the current study, the risk of reporting bias by selective outcome reporting was not considered, since body weight measurements were not always described as being part of the study protocol (Schipper et al., 2018[37]), and we had excluded studies that had no report of body weights. We also did not consider random outcome assessment, blinding of outcome assessor, and incomplete outcome data as domains of quality assessment, since our outcome of interest, that being body weights of animals, was not a primary outcome in the included studies; finally, the maximum value of the quality score of the studies would be 6. When there were disagreements between the reviewers, this was resolved through consensus-oriented discussion or by consulting a third investigator.

### Data collection and synthesis

The following information was carefully extracted from the included studies: First author's name, date of publication, sample size of both treatment and control groups, age, strain (Wistar, Sprague Dawley), health status of the animals (healthy vs type 2 diabetes), dose of NO_3_ (mg L^-1^), the amount of water intake (mL), exposure period (week), mean body weight values (g), and standard deviations of the baseline and final body weights in the treatment and control groups. If a study had several measurements of body weights for a period of less than < 4 weeks, only the last reported values were considered in a separate arm. Furthermore, in studies with multiple treatment groups with various NO_3_ doses, each treated group was considered as a separate arm. When data were presented only graphically, the outcome values were extracted using a simple reproducible method by Adobe Photoshop (Gheibi et al., 2019[11]).

### Statistical methods

Quantitative interpretation of data was based on the weighted mean difference and 95 % confidence interval of the final body weights of intervention and control groups. We also conducted a meta-analysis using the absolute mean differences in body weights relative to the baseline values in both NO_3_-treated and control groups. Further sub-group analyses were conducted to investigate the potential sources of heterogeneity, and to evaluate whether mean differences in the body weights might be affected by the dose of NO_3_, the study duration, or health status of the animals. The treatment effects were accordingly assessed in the following predefined sub-groups including study duration (<4, 4-8 and >8 weeks), dose of NO_3_ (72.94 ≤ or > 72.94 mg L^-1^, the median of the doses), and the health status of the animals (healthy *vs.* type 2 diabetes). 

To identify potential moderators, which could explain the variance in body weights in response to NO_3_ supplementation, random effect meta-regression analyses were also conducted. Using meta-regression, we assessed potential effects in baseline body weights and the age of the animals, dose of NO_3_, study duration and overall exposure to NO_3_ (calculated as doses of NO_3_ × study duration) on the pooled effect sizes. A sensitivity analysis was also conducted by exclusion of the experiments [Hezel, et al. (Hezel et al., 2016[13]) and El-Wakf et al. (El-Wakf et al., 2009[6], 2015[7])] used high doses of NO_3 _(620 and 401 mg L^-1^ d^-1^).

To assess statistical heterogeneity in the meta-analysis, the I^2^ statistic was used according to specific categories (low=25 %, moderate=50 %, high=75 %) (Higgins et al., 2003[14]). The I^2^, a measure of inconsistency between the study results, represents the extent of overlap of the confidence intervals (CIs) of the effect sizes and quantifies the proportion of the observed dispersion (attributed to between-study differences and not to random error) (Higgins et al., 2003[14]; Hooijmans et al., 2014[15]). Although both the fixed- and random-effects models were used to estimate the pooled mean differences in body weights in response to NO_3_, findings from the random-effect models were reported because of existing significant heterogeneity among the studies. 

Potential publication bias was assessed using funnel plots and Eggers's regression test asymmetry for the included studies. Statistical analyses were conducted using STATA version 11 SE (StataCorp LP, TX, USA). All tests were two-tailed, and a probability level <0.05 was considered statistically significant.

## Results

### Study characteristics and quality of included studies 

Of the 175 studies (173 yielded through search of databases + 2 yielded through supplementary hand searching), 130 studies were excluded after initial title/abstract screening, and 45 full-text documents were assessed for eligible criteria. Finally, 11 eligible studies were included in the qualitative and quantitative analyses. Details of the assessment process, exclusion criteria, and number of excluded documents are provided in Figure 1(Fig. 1).

The experiments were conducted between 2004 and 2019 and included a total of 43 intervention (n=395) and 43 control (n=395) arms. All studies reported the effect of NO_3_ on body weight as a secondary outcome and 7 studies were conducted on animal models of type 2 diabetes induced by a combination of high-fat diets and low dose streptozotocin (STZ). Mean baseline body weights of the animals were 281 g (with a range of 38 to 630 g). Duration of the experiments ranged from 1.4 to 25 weeks. Mean dose of NO_3_ treatment was 135 mg L^-1^ (ranged from ~22 to 620 mg L^-1^); eleven experiments used KNO_3_ as the source of NO_3_ while the rest used NaNO_3_. The experimental details of each matched treatment-control group, including age, strain, exposure period, number of animals per experiment, and the dose of NO_3_, are provided in Supplementary Table 1. 

According to SYRCLE's RoB tool, mean quality values of the studies was 1.43; overall, there was a high risk of bias mainly due to uncertainties regarding random housing, blinding of intervention, and allocation concealment. Results of quality assessment of the studies are provided in Supplementary Table 1.

### Meta-analyses, sub-group analyses and meta-regressions 

**Figure 2:**
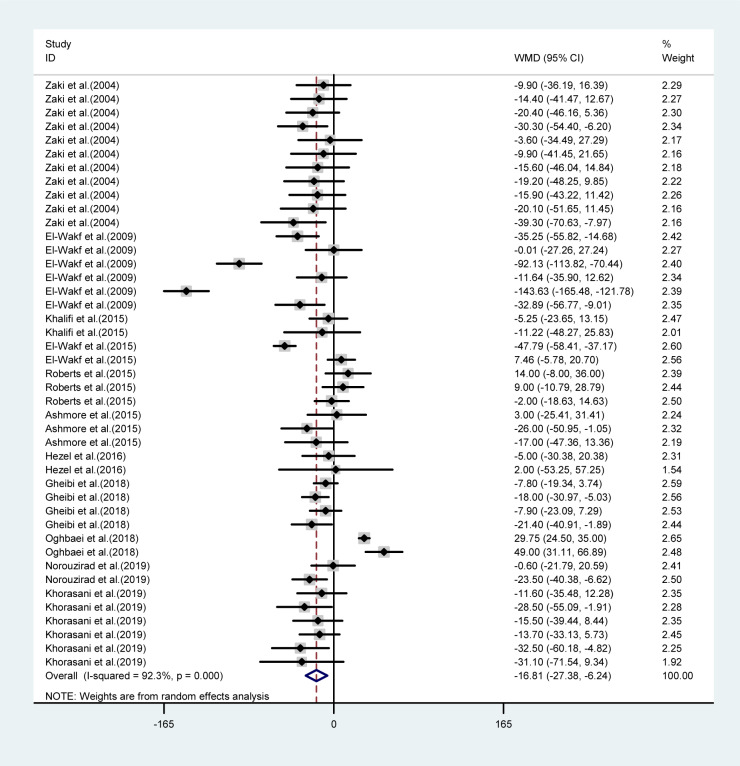
Weighted mean difference of body weight (g) in NO_3_ supplemented groups and controls

Overall, the final body weights were significantly lower in the NO_3_ supplemented groups compared to controls (WMD= -16.8 g, 95 % CI= -27.38, -6.24; *P*=0.002) (Figure 2(Fig. 2); References in Figure 2: Ashmore et al., 2015[2]; El-Wakf et al., 2009[6], 2015[7]; Gheibi et al., 2018[10]; Hezel et al., 2016[13]; Khalifi et al., 2015[21]; Khorasani et al., 2019[22]; Norouzirad et al., 2019[31]; Oghbaei et al., 2018[32]; Roberts et al., 2015[35]; Zaki et al., 2004[40]); after exclusion of 10 experiments within the arms corresponding to high doses of NO_3 _(620 and 401 mg L^-1^ d^-1^), overall mean differences in body weights between the groups decreased by approximately 37 % but remained statistically significant (WMD= -10.1 gr, 95 % CI= -19.0, -1.19, *P*=0.026). 

**Figure 3:**
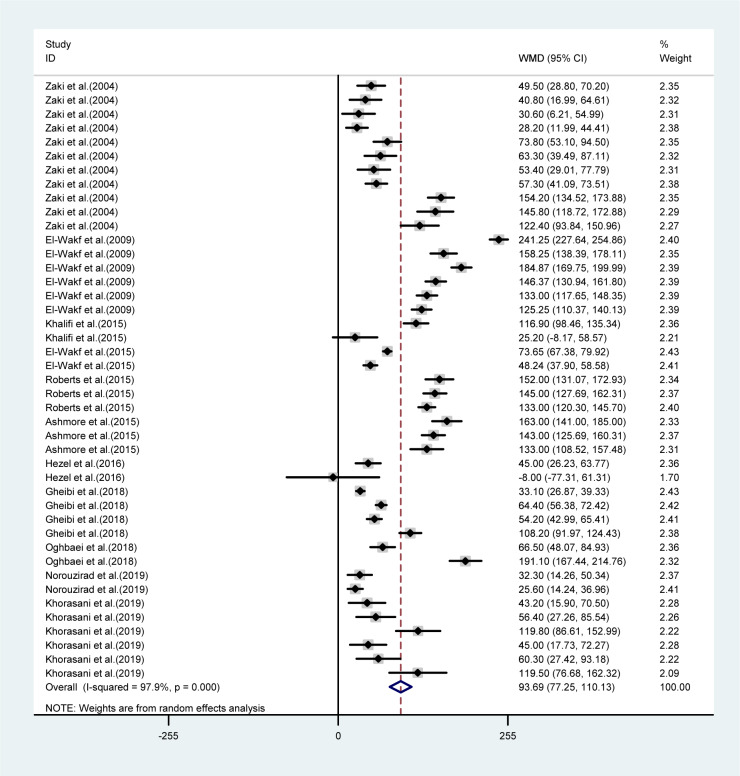
Pooled estimated body weight changes in relation to baseline values in NO_3_ supplemented groups (WMD= 93.69, 95 % CI= 77.25, 110.13, P=0.001)

**Figure 4:**
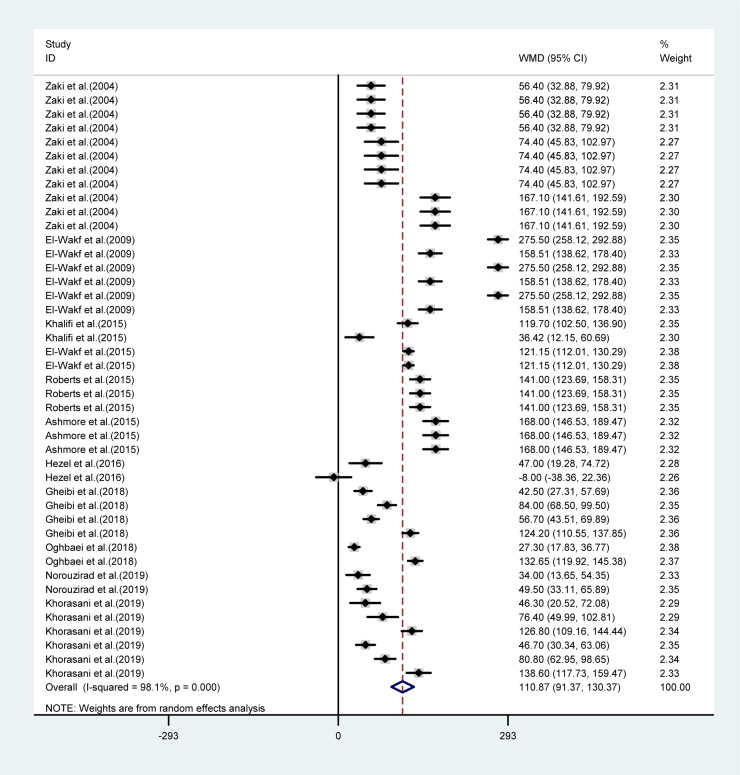
Pooled estimated body weight changes in relation to baseline values in controls (WMD= 110.87, 95 % CI= 91.37, 130.37, P=0.001)

The mean differences in body weights between NO_3_ supplemented groups and controls was greatest in the long-term compared to mid-term (>8 *vs.* 4-8 weeks) study durations (WMD= -23.16 g, 95 % CI=-35.6, -10.7 g *vs.* WMD= -9.19 g, 95 % CI= -17.04, -1.35 g), and was not statistically significant in short-term intervention periods (WMD= 2.34 g, 95 % CI= -13.34, 18.02 g). Highest compared to the lowest doses of NO_3_ (≤ 72.94 *vs.* > 72.94 mg L^-1^ d^-1^) was also related to a greater mean difference in body weights between the groups (WMD= -31.92 g, 95 % CI= -52.90, -10.94 g *vs.* WMD= -8.45 g, 95 % CI= -18.32, 1.41 g). No significant differences in body weights were observed between healthy animals and type 2 diabetic animals (Table 1(Tab. 1)). Mean body weight changes in both NO_3_ supplemented groups and controls are shown in Figures 3 and 4 (References in Figure 3(Fig. 3) and 4(Fig. 4): Ashmore et al., 2015[2]; El-Wakf et al., 2009[6], 2015[7]; Gheibi et al., 2018[10]; Hezel et al., 2016[13]; Khalifi et al., 2015[21]; Khorasani et al., 2019[22]; Norouzirad et al., 2019[31]; Oghbaei et al., 2018[32]; Roberts et al., 2015[35]; Zaki et al., 2004[40]); compared to controls, NO_3_ treated rats gained lower body weights during the study period (WMD= 93.69 g, 95 % CI= 77.25, 110.13 g *vs.* WMD= 110.87 g, 95 % CI= 91.37, 130.37 g).

Meta-regression analyses showed that the mean changes in body weights in the animals were affected by age, baseline body weights of the animals and study duration (Table 1(Tab. 1)); older animals and those with higher baseline body weights showed a lower weight gain in response to daily doses of NO_3_. 

The amount of water intake was similar in both NO_3_-treated rats and controls (WMD= -1.14 mL, 95 % CI= -2.56, 0.29, *P*=0.118) (Supplementary Figure 1). The amount of water intake remained constant in the NO_3_-treated rats during the study period (WMD= 1.11 mL, 95 % CI= -0.51, 2.73, *P*=0.18) (Supplementary Figure 2).

### Publication bias and heterogeneity

An overall symmetric distribution of the studies around the mean effect size observed in the funnel plot indicated a low risk for publication bias. According to results of Egger's regression test, no evidence was observed regarding publication bias among the studies. High heterogeneity values were observed in our meta-analysis models; subgroup analysis (Table 2(Tab. 2)) indicated that experiments with longer duration (> 8 weeks) and those that used higher doses of NO_3_ (> 72.94 mg L^-1^ d^-1^) might be potential sources of heterogeneity for the outcome.

## Discussion

In this systematic review and meta-analysis in male rats, available data on body weights in NO_3_-treated and control groups were collected, evaluated, and analyzed. Meta-analysis of the 43 included animal experiment arms indicated that treatment with inorganic NO_3_ (via drinking water) might be related to a lower weight gain, compared to control animals (93.68 *vs.* 110.87 g, with a mean difference of -16.81 g, 95 % CI= -27.38, -6.24). Effects of NO_3_ on weight gain were greater when higher doses of NO_3_ or longer NO_3 _exposure periods were implemented; further moderator analyses showed that preventive weight-gain capacity of inorganic NO_3_ appears to be affected by age and the baseline body weights of the animals. These data strongly potentiate the hypothesis that introducing inorganic NO_3_ in the diet, acts as a natural anti-obesity agent. Meta-analysis of the amount of water intakes in NO_3_-treated and controls showed that inorganic NO_3_ had not significant effect on regular water intake of the animals. 

Findings of our sensitivity analysis [by exclusion of the experiments that used high doses of NO_3 _to determine its toxic effects (> 400 mg L^-1^ d^-1^) (El-Wakf et al., 2009[6], 2015[7]; Hezel et al., 2016[13])] confirms the robustness of the findings from the main meta-analyses, and suggests that beyond the anti-obesity effects of NO_3 _at high doses (> 400 mg L^-1^), previously explained by an increased protein catabolism and renal protein loss (El-Wakf et al., 2015[7]), inhibition of growth hormone secretion or decreased growth hormone receptors (Jahreis et al., 1987[18], 1991[17]; Mukhopadhyay et al., 2005[30]), NO_3_ at doses close to those concentrations can be achieved through the usual dietary intakes, can also be considered as an anti-obesity agent. Such doses are also comparable to the amount of endogenous NO_3_ derived from eNOS under normal condition in mice (Carlstrom et al., 2010[4]), and are also sufficient for induction of NO-like activity in both human and animals (Weitzberg and Lundberg, 2013[39]; Lundberg et al., 2018[25]). 

At physiological levels (PM to nM), NO regulates blood flow and vascularization of adipose tissue in a soluble guanylyl cyclase (sGC) and cyclic guanosine monophosphate (cGMP) dependent manner (Jobgen et al., 2006[20]). NO regulates adipose tissue metabolism through activation of the peroxisome proliferator activated receptor γ (PPARγ), uncoupling protein-1 (UCP-1), stimulation of mitochondrial biogenesis, regulation of adipogenesis, lipolysis and insulin-stimulated glucose uptake (McGrowder et al., 2006[28]; Knott and Bossy-Wetzel, 2010[23]). Although the underlying mechanisms explaining the anti-obesity effects of oral NO_3_ loading are not fully understood, exogenous NO_3_ appears to effectively mimic physiologic properties of NO in adipose tissue; for example, administration of NO_3_ exerts anti-obesity effects via induction of adipose tissue browning (switching from white adipose tissue-like to brown-like phenotypes) (Roberts, 2015[34]; Roberts et al., 2015[35]). Inorganic NO_3_ (as NaNO_3_ at doses of 0.35, 0.7, and 1.4 mmol L^-1^ of drinking water) dose-dependently increases brown adipocyte-specific gene expression e.g. UCP-1, peroxisome proliferator-activated receptor gamma coactivator-1α (PGC-1α), cytochrome *c*, carnitine palmitoyltransferase I (CPT1) and acyl-CoA dehydrogenase in white adipose tissue (Roberts et al., 2015[35]). NO_3_ treatment also induces mitochondrial biogenesis (measured as increased citrate synthase activity, a marker of mitochondrial density) (Roberts et al., 2015[35]), an important target of obesity treatment (Hey-Mogensen and Clausen, 2017[12]). Since almost all studies reported similar amount of food intakes among the NO_3_-treated groups and controls (Ashmore et al., 2015[2]; Khalifi et al., 2015[21]; Roberts et al., 2015[35]; Hezel et al., 2016[13]; Gheibi et al., 2018[10]; Khorasani et al., 2019[22]; Norouzirad et al., 2019[31]), inorganic NO_3_ administration does not seem to exert its anti-obesity effects through modulation of appetite and total calorie intakes.

One important limitation of the current meta-analysis was the considerable heterogeneity between the included studies. The high-heterogeneity of the estimated mean difference of body weights may be attributed to the different experimental designs of the included studies (i.e. various NO_3_ doses and wide ranges of exposure period), and variation in terms of age and baseline body weights of the animals. Although use of sub-group analyses and meta-regressions gives us new insight into the potential sources of heterogeneity, other factors including dietary background of the animals or the amount of food intakes may be involved, which we could not explore them in this study due to lack of enough reported data. 

Since part of the heterogeneity between animal studies is caused by differences in biological study characteristics including species, sex, and intervention schedule (Hooijmans et al., 2014[15]), we defined a set of strict inclusion and exclusion criteria and restricted our systematic search to a specific species (rats), sex (males), and specific rout of intervention (drinking water). Such an approach enabled us in making only sensible comparisons, addressing a clear question and providing meaningful effect sizes. On the other hand, use of random-effect models that provide wider CIs compared with that of a fixed-effect estimate, makes our estimated effect sizes to be more reliable since this approach considers some possible variations in the true effect size besides chance alone (Hooijmans et al., 2014[15]). 

To the best of our knowledge, this is the first systematic review and meta-analysis to explore the potential effects of inorganic NO_3_ exposure in animal models on body weight. Considering high heterogeneity of the included studies and the use of random-effect models for estimation of the effect sizes, we cannot be quiet certain that inorganic NO_3_ has preventive weight-gain capacity, however, we can conclude that on average NO_3_-treated animals have lower body weights, but the true treatment effect may differ between settings. However, such preliminary data is warranted to be further evaluated and confirmed through a well-defined, clear hypothesis, and well-designed experiments. Inorganic NO_3_ may be considered as a promising dietary-based therapeutic approach for management of obesity.

## Notes

Zahra Bahadoran and Sajad Jeddi contributed equally as first authors.

## Supplementary Material

Supplementary information

## Figures and Tables

**Table 1:**
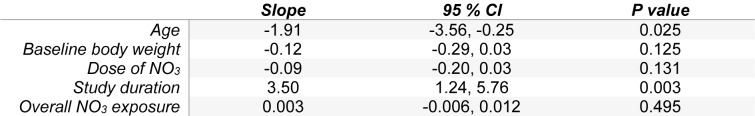
Meta-regression of potential moderators of body weight changes in response to NO_3_ treatment

**Table 2:**
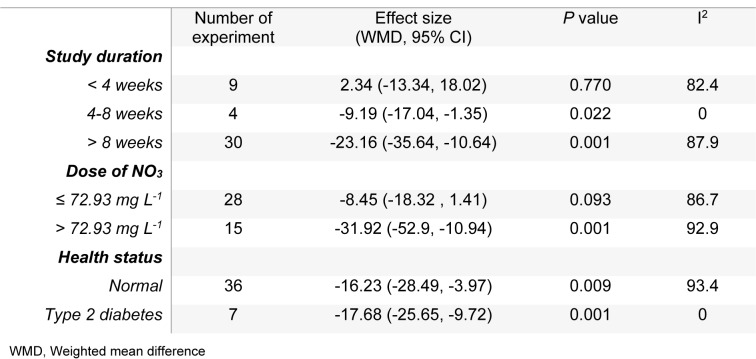
Meta-analysis of mean difference of body weight in NO_3_ supplemented groups and controls according to study duration, dose of NO_3_ and health status
