# Supplementary material for: Inorganic nitrate, a natural anti-obesity agent: A systematic review and meta-analysis of animal studies
Source: EXCLI J. 2020 Jul 6;19:972–83. doi: 10.17179/excli2020-2515 (PMC7415936; doi:10.17179/excli2020-2515)
Supplement: Supplementary information [file EXCLI-19-972-s-001.pdf]

EXCLI Journal 2020;19:972-983 – ISSN 1611-2156
Received: June 07, 2020, accepted: June 30, 2020, published: July 06, 2020

Review article:

INORGANIC NITRATE, A NATURAL ANTI-OBESITY AGENT: A SYSTEMATIC REVIEW AND META-ANALYSIS OF ANIMAL STUDIES

Zahra Bahadoran[1#], Sajad Jeddi[2#], Sevda Gheibi[2,3], Parvin Mirmiran[4], Khosrow Kashfi[5], Asghar Ghasemi[2*]

[1] Nutrition and Endocrine Research Center, Research Institute for Endocrine Sciences, Shahid Beheshti University of Medical Sciences, Tehran, Iran
[2] Endocrine Physiology Research Center, Research Institute for Endocrine Sciences, Shahid Beheshti University of Medical Sciences, Tehran, Iran
[3] Department of Clinical Sciences in Malmö, Unit of Molecular Metabolism, Lund University Diabetes Centre, Clinical Research Center, Malmö University Hospital, Lund University, Malmö, Sweden
[4] Department of Clinical Nutrition and Human Dietetics, Faculty of Nutrition Sciences and Food Technology, National Nutrition and Food Technology Research Institute, Shahid Beheshti University of Medical Sciences, Tehran, Iran
[5] Department of Molecular, Cellular and Biomedical Sciences, Sophie Davis School of Biomedical Education, City University of New York School of Medicine, New York, NY 10031, USA

# These authors contributed equally to this paper.

* **Corresponding author:** Asghar Ghasemi, Endocrine Physiology Research Center, Research Institute for Endocrine Sciences, Shahid Beheshti University of Medical Sciences, Tehran, Iran, No. 24, Sahid-Erabi St, Yemen St, Chamran Exp, Tehran, Iran. P.O.Box: 19395-4763, Phone: +98 (21) 224 32 500, Fax: +98 (21) 224 16 264, 224 02 463, E-mail address: Ghasemi@endocrine.ac.ir

http://dx.doi.org/10.17179/excli2020-2515

This is an Open Access article distributed under the terms of the Creative Commons Attribution License (http://creativecommons.org/licenses/by/4.0/).

EXCLI Journal 2020;19:972-983 – ISSN 1611-2156
Received: June 07, 2020, accepted: June 30, 2020, published: July 06, 2020

Supplementary Table 1: Characteristics of the included studies

Study	Animal	Study duration (week)	Doses of NO_3 (mg L^{-1} drinking water)	Change of body weight	Food intakes	Water consumption	Quality assess-ment
**Zaki et al. 2004**	Male healthy Wistar rats	21.43	30.69, 61.38, 92.07, 306.09	↓	NR	NR	1
**El-Wakf et al. 2009 and 2015**	Male healthy Wistar rats	17.14	72.94, 182.35, 401.17	↓	NR	NR	1
**Roberts et al. 2015**	Male healthy Wistar rats	2.57	21.88, 43.76 and 87.53	↔	↔	↔	1
**Ashmore et al. 2015**	Male healthy Wistar rats	2.57	21.88, 43.76 and 87.53	↔	↔	↔	1
**Oghbaei et al. 2018**	Male healthy and diabetic Wistar rats	8.57	72.94	↑	NR	NR	2
**Khorasani et al. 2019**	Male healthy and diabetic Wistar rats	15	72.94	↓	↔	↑	2
**Norouzirad et al. 2019**	Male healthy and diabetic Wistar rats	5	72.94	↓in diabetic rats	↔	↔	2
**Gheibi et al. 2018**	Healthy and dia-betic male Wistar rats	8	72.94	↓	↔	↔	2
**Hezel et al. 2016**	Male young and old Sprague Dawley rats	2	619.99	↑ in male young rats	↔	NR	2
**Khalifi et al. 2015**	Healthy and diabetic male Wistar rats	8	72.94	↔	↔	↔	1

EXCLI Journal 2020;19:972-983 – ISSN 1611-2156
Received: June 07, 2020, accepted: June 30, 2020, published: July 06, 2020

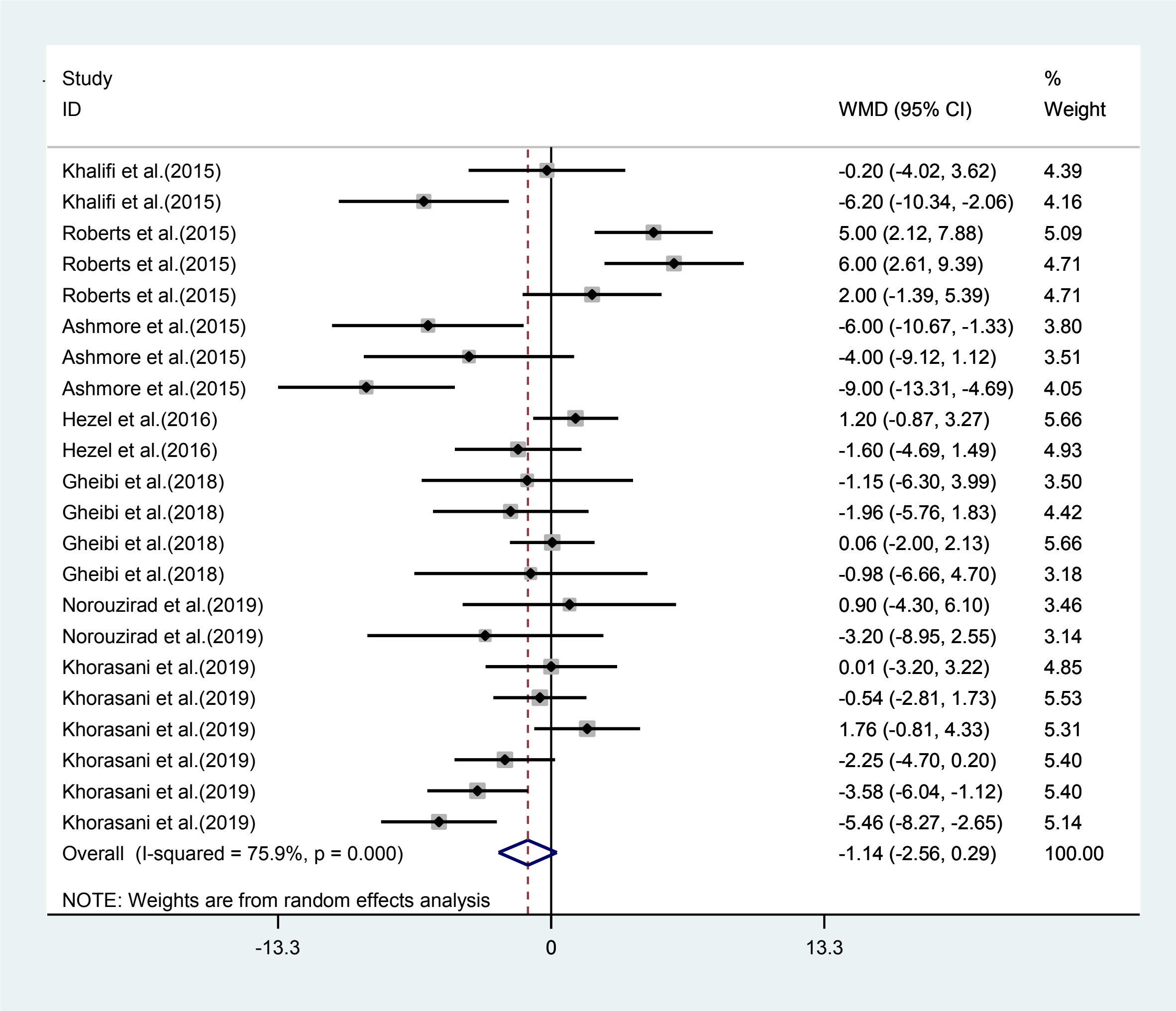

Supplementary Figure 1: Pooled estimated water intakes in NO_3-treated rats and controls (final amount of water intakes) (WMD= -1.14 mL, 95 % CI= -2.56, 0.29, P=0.118)

EXCLI Journal 2020;19:972-983 – ISSN 1611-2156
Received: June 07, 2020, accepted: June 30, 2020, published: July 06, 2020

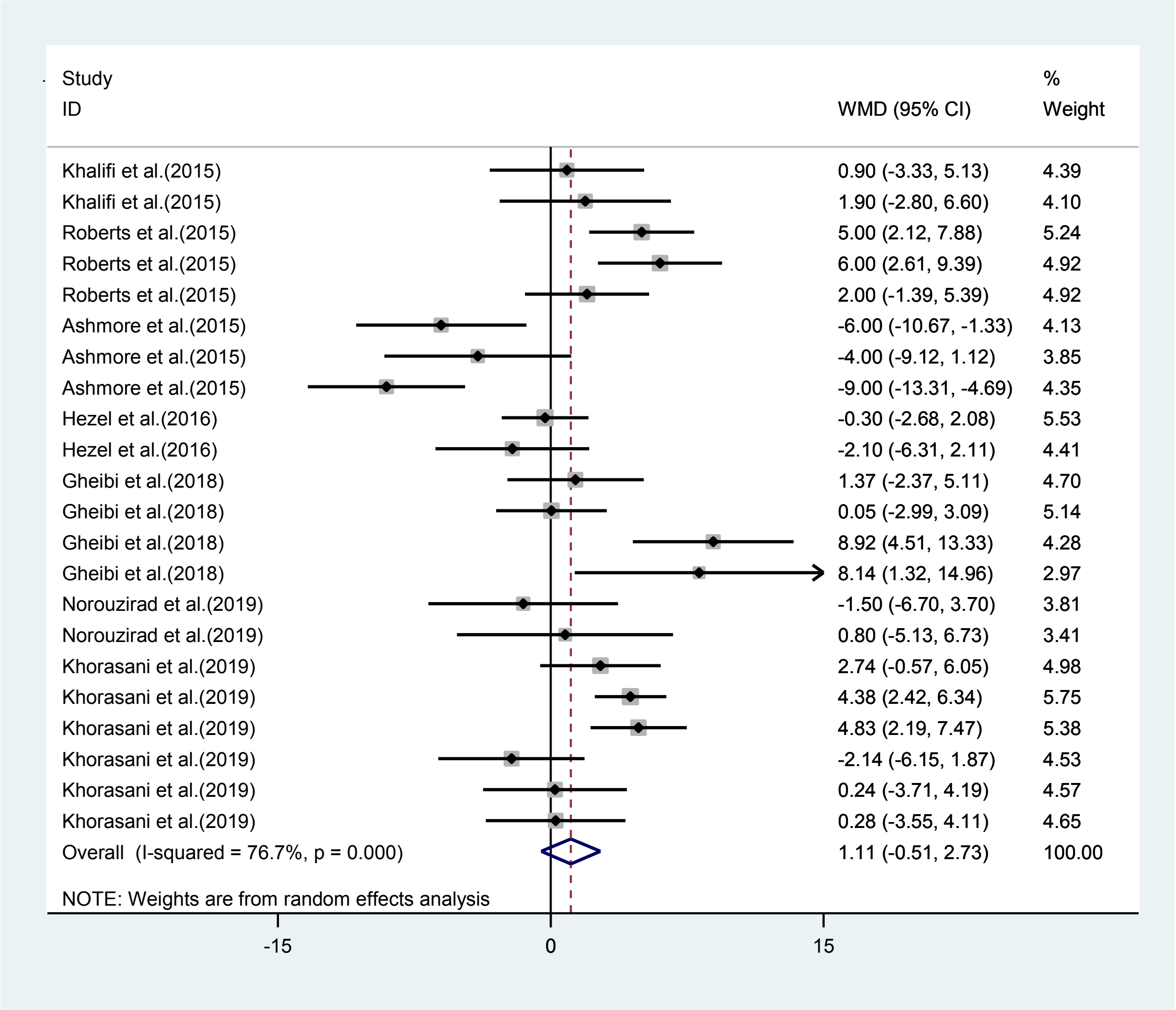

Supplementary Figure 2: Pooled estimated amount of water intakes in NO_3-treated compared to baseline values (WMD= 1.11 mL, 95 % CI= -0.51, 2.73, P=0.18)
